# Complete genome sequence of *Bacillus velezensis* Tcba05, a biocontrol strain for multiple plant diseases in Taiwan

**DOI:** 10.1128/mra.00212-25

**Published:** 2025-08-25

**Authors:** Shih-Hsun Walter Hung, Chia-Ho Liu, Chien-Chih Kuo, Chieh-Chen Huang, Chih-Horng Kuo

**Affiliations:** 1 Department of Life Sciences, National Chung Hsing University 34916, Taichung, Taiwan; 2 Institute of Plant and Microbial Biology, Academia Sinica 38017, Taipei, Taiwan; 3 Advanced Plant and Food Crop Biotechnology Center, National Chung Hsing University 34916, Taichung, Taiwan; 4 Innovation and Development Center of Sustainable Agriculture, National Chung Hsing University 34916, Taichung, Taiwan; 5 Taichung District Agricultural Research and Extension Station, Ministry of Agriculture, Taichung, Taiwan; 6 Biotechnology Center, National Chung Hsing University 34916, Taichung, Taiwan; University of Strathclyde, Glasgow, United Kingdom

**Keywords:** genome, *Bacillus velezensis*, *Bacillus amyloliquefaciens*, biological control agent (BCA), plant growth-promoting bacteria (PGPB)

## Abstract

In this work, we report the complete genome sequence of *Bacillus velezensis* strain Tcba05. This strain is a potent biocontrol agent against multiple plant diseases in Taiwan, including asparagus bean *Fusarium* wilt and cabbage black rot. The assembly consists of a single circular chromosome of 4,027,462 bp with no plasmids.

## ANNOUNCEMENT

The strain Tcba05 was isolated from the rhizospheric soil of a citrus tree in Taiwan (coordinates: 23.725745, 120.852890) by the Taichung District Agricultural Research and Extension Station on 20 June 2012 (1). It was initially identified as *Bacillus amyloliquefaciens* and exhibited biological control activity against *Fusarium oxysporum* and *Xanthomonas campestris* (1, 2). Here, we report its genome and revise its taxonomic classification.

A hybrid *de novo* assembly strategy was employed, following previous studies (3–6). Unless otherwise stated, kits were used per manufacturer instructions, and bioinformatics tools were applied with default settings.

The strain was revived from a −80°C glycerol stock in Chieh-Chen Huang’s laboratory by streaking onto LB (279110; BD Difco, USA) agar and incubated at 30°C for 48 h. A single colony was picked and grown in LB broth at 30°C with shaking (200 rpm) for 48 h. The same inoculum was used for both sequencing platforms. For Illumina, DNA was extracted using the Wizard Genomic DNA Purification Kit (A1120; Promega, USA) and processed using the NEB Library Preparation Kit (07961880001; Roche, Switzerland). Sequencing on NovaSeq X Plus produced 13,589,230 151 bp paired-reads (Q30: 91.82%); no trimming or filtering was conducted. For Oxford Nanopore Technologies (ONT), DNA was extracted using the Nanobind CBB Kit (102-301-900; PacBio, USA). The ONT Ligation Sequencing Kit (SQK-LSK109) and Expansion Kit (EXP-NBD104) were used without shearing for library construction. Sequencing based on MinION (R9.4.1 flow cell; FLO-MIN106) and basecalling with Guppy v6.5.7 in super-accurate mode produced 23,700 raw reads (N50: 33,990 bp; total: 567.5 Mb). Applying a 30,000 bp length cutoff yielded 6,961 filtered reads (N50: 47,236 bp; total: 326.8 Mb).

Assembly using Unicycler v0.5.0 (7) with all Illumina reads and filtered ONT reads produced one closed circular chromosomal contig (4,027,462 bp; 46.5% GC), starting at *dnaA*. Validation involved read mapping with BWA v0.7.17 (8) and Minimap2 v2.15 (9), automated checking with SAMtools v1.9 (10), and manual inspection in IGV v2.11.1 (11). Four mismatches were corrected, yielding an error rate below 10^−6^. The Illumina and filtered ONT reads provided 509- and 81-fold coverage, respectively.

Annotation was performed by the National Center for Biotechnology Information (NCBI) using the Prokaryotic Genome Annotation Pipeline (PGAP) v6.9 (12). Nine complete rRNA operons, 86 tRNA genes, five ncRNAs, and 3,825 protein-coding genes were annotated. Based on average nucleotide identity (ANI) analysis using fastANI v1.34 (13), Tcba05 shared 92.31% alignment fraction and 97.48% ANI with *Bacillus velezensis* KCTC 13012^T^ (GCF_001267695.1), which belongs to the operational group *B. amyloliquefaciens* (14). According to the 95%–96% ANI threshold suggested for prokaryotic species delineation (15, 16), Tcba05 was reclassified as *B. velezensis*.

The benchmarking universal single-copy ortholog (BUSCO) analysis v5.1.2 (17) against the Bacillales data set was performed using gVolante v2.0.0 (18). The result identified 449 complete BUSCOs (99.8%), one fragmented (0.2%), and none missing. This assessment, together with the chromosomal contig being circular, supported that this genome assembly is complete.

## Data Availability

This genome project has been deposited in NCBI under the BioProject accession number PRJNA1191524. Metadata associated with this strain have been provided in BioSample SAMN45085276. The Illumina and ONT raw reads have been deposited in NCBI Sequence Read Archive under accession numbers SRX27637767 and SRX27637766, respectively. The genome sequence has been deposited in GenBank under accession number CP175541.

## References

[1] Kuo CC, Chen CW, Liao CT, Chen WL, Tsai YF. 2014. Development and application of Bacillus amyloliquefaciens on plant diseases biocontrol. Special Issue of Taichung District Agricultural Research and Extension Station 121:69–86.

[2] Kuo CC, Chuang TH, Chen CW, Liao CT. 2017. The evaluated of functional microbial agents application for the cabbage cultivation and black rot disease control. Bulletin of Taichung District Agricultural Research and Extension Station 137:37–57.

[3] Hung S-HW, Chiu M-C, Huang C-C, Kuo C-H. 2022. Complete genome sequence of Curtobacterium sp. C1, a beneficial endophyte with the potential for in-plant salinity stress alleviation. Mol Plant Microbe Interact 35:731–735. doi:10.1094/MPMI-01-22-0027-A 35819348

[4] Hung SH, Wu IC, Huang CC, Kuo CH. 2023. Complete genome sequence of Erythrobacteraceae bacterium WH01K, a strain isolated from corals (Acropora sp.) in Taiwan. Microbiol Resour Announc 12:e0083023. doi:10.1128/MRA.00830-23 37933969 PMC10720573

[5] Hung SH, Yeh PH, Huang TC, Huang SY, Wu IC, Liu CH, Lin YH, Chien PR, Huang FC, Ho YN, Kuo CH, Hwang HH, Chiang EP, Huang CC. 2024. A cyclic dipeptide for salinity stress alleviation and the trophic flexibility of endophyte provide insights into saltmarsh plant–microbe interactions. ISME Communications 4:ycae041. doi:10.1093/ismeco/ycae041 38707842 PMC11070113

[6] Hung SHW, Yu MY, Liu CH, Huang TC, Peng JH, Jang NY, Kuo CH, Yang YL, Ho YN, Chiang EPI, Hwang HH, Huang CC. 2024. Endophytic pyrroloquinoline quinone enhances banana growth and immunity against Fusarium wilt for plant-microbe mutualisms. bioRxiv. doi:10.1101/2024.08.20.608638 PMC13133254 42062822

[7] Wick RR, Judd LM, Gorrie CL, Holt KE. 2017. Unicycler: resolving bacterial genome assemblies from short and long sequencing reads. PLoS Comput Biol 13:e1005595. doi:10.1371/journal.pcbi.1005595 28594827 PMC5481147

[8] Li H, Durbin R. 2009. Fast and accurate short read alignment with Burrows-wheeler transform. Bioinformatics 25:1754–1760. doi:10.1093/bioinformatics/btp324 19451168 PMC2705234

[9] Li H. 2018. Minimap2: pairwise alignment for nucleotide sequences. Bioinformatics 34:3094–3100. doi:10.1093/bioinformatics/bty191 29750242 PMC6137996

[10] Li H, Handsaker B, Wysoker A, Fennell T, Ruan J, Homer N, Marth G, Abecasis G, Durbin R. 2009. 1000 Genome project data processing subgroup. Bioinformatics 25:2078–2079. doi:10.1093/bioinformatics/btp352 19505943 PMC2723002

[11] Robinson JT, Thorvaldsdóttir H, Winckler W, Guttman M, Lander ES, Getz G, Mesirov JP. 2011. Integrative genomics viewer. Nat Biotechnol 29:24–26. doi:10.1038/nbt.1754 21221095 PMC3346182

[12] Tatusova T, DiCuccio M, Badretdin A, Chetvernin V, Nawrocki EP, Zaslavsky L, Lomsadze A, Pruitt KD, Borodovsky M, Ostell J. 2016. NCBI prokaryotic genome annotation pipeline. Nucleic Acids Res 44:6614–6624. doi:10.1093/nar/gkw569 27342282 PMC5001611

[13] Guindon S, Gascuel O. 2003. A simple, fast, and accurate algorithm to estimate large phylogenies by maximum likelihood. Syst Biol 52:696–704. doi:10.1080/10635150390235520 14530136

[14] Fan B, Blom J, Klenk HP, Borriss R. 2017. Bacillus amyloliquefaciens, Bacillus velezensis, and Bacillus siamensis form an “Operational Group B. amyloliquefaciens” within the B. subtilis species complex. Front Microbiol 8:22. doi:10.3389/fmicb.2017.00022 28163698 PMC5247444

[15] Jain C, Rodriguez-R LM, Phillippy AM, Konstantinidis KT, Aluru S. 2018. High throughput ANI analysis of 90K prokaryotic genomes reveals clear species boundaries. Nat Commun 9:5114. doi:10.1038/s41467-018-07641-9 30504855 PMC6269478

[16] Riesco R, Trujillo ME. 2024. Update on the proposed minimal standards for the use of genome data for the taxonomy of prokaryotes. Int J Syst Evol Microbiol 74:006300. doi:10.1099/ijsem.0.006300 38512750 PMC10963913

[17] Manni M, Berkeley MR, Seppey M, Simão FA, Zdobnov EM. 2021. BUSCO update: novel and streamlined workflows along with broader and deeper phylogenetic coverage for scoring of eukaryotic, prokaryotic, and viral genomes. Mol Biol Evol 38:4647–4654. doi:10.1093/molbev/msab199 34320186 PMC8476166

[18] Nishimura O, Hara Y, Kuraku S. 2019. Evaluating genome assemblies and gene models using gVolante. Methods Mol Biol 1962:247–256. doi:10.1007/978-1-4939-9173-0_15 31020565

